# Supplementary material for: The Evolution and Characterization of the RNA Interference Pathways in Lophotrochozoa
Source: Genome Biol Evol. 2024 May 7;16(5):evae098. doi: 10.1093/gbe/evae098 (PMC11114477; doi:10.1093/gbe/evae098)
Supplement: evae098_Supplementary_Data [file evae098_supplementary_data.zip › Supplementary_materials.zip!/selected_miRNAs/NoGe_selected_miRNAs/mirdeep_pdf_files/NMRB01000188.1_Notospermus_geniculatus_strain_Ushimado_scaffold188,_whole_genome_shotgun_sequence_6824.pdf]

Provisional ID	:	NMRB01000188.1_Notospermus_geniculatus_strain_Ushimado_scaffold188,_whole_genome_shotgun_sequence_6824
Score total	:	1876.1
Score for star read(s)	:	3.9
Score for read counts	:	1867.6
Score for mfe	:	1.6
Score for randfold	:	0
Score for cons. seed	:	3
Total read count	:	3675
Mature read count	:	3379
Loop read count	:	4
Star read count	:	292

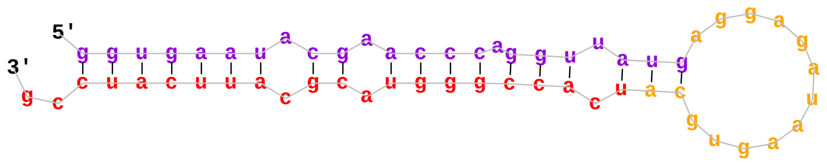

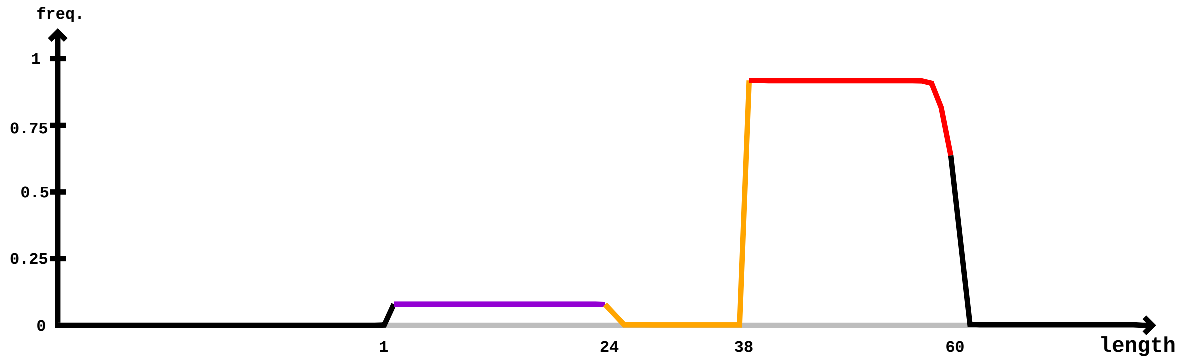


```
                                      Star                                   Mature
5'- cccauggaauaagaaacaacgcaugucacuuccggugaauacgaacccagguuaugaggagauaagugcaucaccggguacgcauucauccgggugugg cugugcauuucau   -3'   obs
    cccauggaauaagaaacaacgcaugucacuuccggugaauacgaacccagguuaugaggagauaagugcaucaccggguacgcauucauccggguguggcugugcauuucau         exp
    ...(((((((..........(((((((((.((((((((((.((.((((.(((.(((............))).))))))).)).))))).))))).))))).)))))))))))    reads   mm   sample
    ......................................................ugaggagauaagAgcauc........................................    4       1    Fm3
    ......................................................................ucaccggguacgcauucCu.......................    1       1    Fm3
    ......................................................................ucaccggguacgcauucau.......................    19      0    Fm3
    ......................................................................ucaccggguaUgcauucauc......................    10      1    Fm3
    ......................................................................ucaccggguacgcauuGauc......................    1       1    Fm3
    ......................................................................ucaccggguacgcauucUuc......................    1       1    Fm3
    ......................................................................ucaccggguacgcauucauc......................    176     0    Fm3
    ......................................................................ucaccggguacgcauuAauc......................    1       1    Fm3
    ......................................................................Acaccggguacgcauucauc......................    1       1    Fm3
    ......................................................................ucaccggguacgcauucauU......................    3       1    Fm3
    ......................................................................ucaccggguacgcauucGuc......................    1       1    Fm3
    ......................................................................ucaGcggguacgcauucauc......................    1       1    Fm3
    ......................................................................ucaAcggguacgcauucaucc.....................    1       1    Fm3
    ......................................................................ucaccggguacgcauucCucc.....................    2       1    Fm3
    ......................................................................ucaccggguaGgcauucaucc.....................    1       1    Fm3
    ......................................................................ucUccggguacgcauucaucc.....................    1       1    Fm3
    ......................................................................ucaccggguacgcauucGucc.....................    1       1    Fm3
    ......................................................................ucaccggguacgcauuAaucc.....................    1       1    Fm3
    ......................................................................uAaccggguacgcauucaucc.....................    1       1    Fm3
    ......................................................................ucaccggguacUcauucaucc.....................    1       1    Fm3
    ......................................................................ucaccggguacgcauucUucc.....................    1       1    Fm3
    ......................................................................ucaccggguacgcauucaucc.....................    345     0    Fm3
    ......................................................................ucaccggguacgcauucaucU.....................    20      1    Fm3
    ......................................................................ucaccggguacgcauucaucA.....................    5       1    Fm3
    ......................................................................ucGccggguacgcauucauccg....................    2       1    Fm3
    ......................................................................ucaccggguacgcauucUuccg....................    1       1    Fm3
    ......................................................................ucaccggguaGgcauucauccg....................    1       1    Fm3
    ......................................................................ucCccggguacgcauucauccg....................    1       1    Fm3
    ......................................................................ucaccggguacgcauuUauccg....................    1       1    Fm3
    ......................................................................ucaccgggCacgcauucauccg....................    1       1    Fm3
    ......................................................................ucaccggguacgcauucauccA....................    5       1    Fm3
    ......................................................................ucaccggguacgcauuGauccg....................    1       1    Fm3
    ......................................................................ucaccggguacgcauucGuccg....................    4       1    Fm3
```

Star Mature

```
cccauggaauaagaaacaacgcaugucacuuccggugaauacgaacccagguuaugaggagauaagugcaucaccggguacgcauucauccggguguggcugugcauuucau
......................................................................ucUccggguacgcauucauccg....................   2     1   Fm3
......................................................................ucaccggguacgcauucaAccg....................   1     1   Fm3
......................................................................ucaccggguacgcauucauUcg....................   1     1   Fm3
......................................................................ucaccggguaUgcauucauccg....................   2     1   Fm3
......................................................................ucaccgAguacgcauucauccg....................   1     1   Fm3
......................................................................ucaccggguacgcauucauccU....................   4     1   Fm3
......................................................................ucaccggguacgcauucCuccg....................   1     1   Fm3
......................................................................ucaccggguacUcauucauccg....................   1     1   Fm3
......................................................................ucaccggguaAgcauucauccg....................   3     1   Fm3
......................................................................ucaccggguacgcauucauccg....................   1061  0   Fm3
......................................................................ucaccggguacgcauuAauccg....................   9     1   Fm3
......................................................................ucacUggguacgcauucauccg....................   2     1   Fm3
......................................................................ucaccggguacgcauucauccgA...................   5     1   Fm3
......................................................................ucaccggguaAgcauucauccgg...................   1     1   Fm3
......................................................................uAaccggguacgcauucauccgg...................   1     1   Fm3
......................................................................ucaccggguacgcauucUuccgg...................   1     1   Fm3
......................................................................ucaccggguacgcauuAauccgg...................   1     1   Fm3
......................................................................ucaccggguacgcauucauccgU...................   32    1   Fm3
......................................................................ucaccggguacgcauucauccgg...................   208   0   Fm3
......................................................................ucaccggguacgcauucauccggA..................   3     1   Fm3
.............................................................................................guguggcugugcauuuca.   8     0   Fm3

.................................ggugaauacgaacccagguuaug........................................................   2     0   Mm3
.................................ggugaauacgaacccaggCuaugagga....................................................   1     1   Mm3
......................................................................ucaccggguacgcauucau.......................   2     0   Mm3
......................................................................ucaccggguacgcauucauc......................   18    0   Mm3
......................................................................ucGccggguacgcauucaucc.....................   1     1   Mm3
......................................................................ucaccggguacgcauucaucU.....................   1     1   Mm3
......................................................................ucaccggguacgcauuGaucc.....................   1     1   Mm3
......................................................................ucaccggguacgcauucaucc.....................   27    0   Mm3
......................................................................ucaccggguacgcauuAauccg....................   1     1   Mm3
......................................................................ucaccggguacgcauucauccU....................   1     1   Mm3
......................................................................ucaccggguaUgcauucauccg....................   1     1   Mm3
......................................................................ucaccggguacgcauucaucUg....................   1     1   Mm3
......................................................................ucaccggguacgcauucCuccg....................   1     1   Mm3
......................................................................uGaccggguacgcauucauccg....................   1     1   Mm3
......................................................................ucaccggguacgcauucauccg....................   120   0   Mm3
......................................................................ucaccgggCacgcauucauccg....................   1     1   Mm3
......................................................................ucaccggguacgcauucauccgU...................   2     1   Mm3
......................................................................ucaccggguaAgcauucauccgg...................   1     1   Mm3
......................................................................ucaccggguacgcauucauccgg...................   39    0   Mm3
......................................................................ucaccggguacgcauucauccgC...................   1     1   Mm3
......................................................................ucaccggguacgcauucauccgA...................   1     1   Mm3

.................................ggugaauacgaacccagAuuaug........................................................   2     1   Fm2
.................................ggugaauacgaacccagguuaug........................................................   24    0   Fm2
.................................ggugaauacgaacccaggCuaug........................................................   1     1   Fm2
......................................................................ucaccggguacgcauucaucc.....................   10    0   Fm2
......................................................................ucaccggguacgcauucauccg....................   39    0   Fm2
......................................................................ucaccggguacgcauucGuccg....................   1     1   Fm2
......................................................................ucaccggguacgcauucauccgU...................   10    1   Fm2

................................cggugaauacgaacccagguuau.........................................................   1     0   Fm1
................................cggugaauacgaacccagguuaug........................................................   3     0   Fm1
.................................ggugaauacgaacccagguua..........................................................   4     0   Fm1
.................................ggugaauacgaacccagguuau.........................................................   4     0   Fm1
.................................ggugaauacgaacccagguuaug........................................................   192   0   Fm1
.................................ggugaauacgaacccagUuuaug........................................................   4     1   Fm1
.................................ggugaauacgaacccagCuuaug........................................................   1     1   Fm1
.................................ggugaauacgaacccagAuuaug........................................................   3     1   Fm1
.................................ggugaauacgaacUcagguuaug........................................................   1     1   Fm1
.................................ggugaauacgaacccagguuauA........................................................   5     1   Fm1
.................................ggugaauacgaacccagguuaugU.......................................................   40    1   Fm1
.................................ggugaauacgaacccagguuaugaggagauaagugca..........................................   1     0   Fm1
......................................................................ucaccggguacgcauucU........................   3     1   Fm1
......................................................................ucaccggguacgcauucUu.......................   1     1   Fm1
......................................................................ucaccggguacgcauucau.......................   4     0   Fm1
......................................................................ucaccggguacgcauucauc......................   40    0   Fm1
......................................................................ucaccggguacgcauucauU......................   2     1   Fm1
......................................................................ucaccggguacgcauucaucc.....................   52    0   Fm1
```

```
                                 Star                                  Mature
cccauggaauaagaaacaacgcaugucacuuccggugaauacgaacccagguuaugaggagauaagugcaucaccggguacgcauucauccggguguggcugugcauuucau
......................................................................ucaccggguacgcauucUucc.....................   1    1   Fm1
......................................................................ucaccggguacgcauucaucU.....................   1    1   Fm1
......................................................................ucaccggguacgcauucaucA.....................   1    1   Fm1
......................................................................ucaccggguacgcauuAauccg....................   2    1   Fm1
......................................................................ucaccggguacgcauucGuccg....................   1    1   Fm1
......................................................................uAaccggguacgcauucauccg....................   1    1   Fm1
......................................................................ucaccggguacgcauucUuccg....................   1    1   Fm1
......................................................................ucaccggguaAgcauucauccg....................   1    1   Fm1
......................................................................ucaccggguacgcauucauccg....................   190  0   Fm1
......................................................................ucaccggguaGgcauucauccgg...................   1    1   Fm1
......................................................................ucaccggguacgcauucCuccgg...................   1    1   Fm1
......................................................................ucaccggguacgcauucauccgA...................   1    1   Fm1
......................................................................ucaccCgguacgcauucauccgg...................   1    1   Fm1
......................................................................ucaccggguacgcauucauccgU...................   24   1   Fm1
......................................................................ucaccggguacgcauucUuccgg...................   1    1   Fm1
......................................................................ucaccggguacgcauucauccgg...................   189  0   Fm1
......................................................................ucaccggguacgcauucauccggA..................   2    1   Fm1

......................................................................ucaccggguacgcauucau.......................   4    0   Mm1
......................................................................ucaccggguacgcauuAauc......................   1    1   Mm1
......................................................................ucaccggguaGgcauucauc......................   1    1   Mm1
......................................................................ucaccggguacgcauucauc......................   40   0   Mm1
......................................................................ucaccggguacgcauuUauc......................   1    1   Mm1
......................................................................ucaccgUguacgcauucauc......................   1    1   Mm1
......................................................................ucaccggguacgcauucaucc.....................   106  0   Mm1
......................................................................ucaccgAguacgcauucaucc.....................   1    1   Mm1
......................................................................ucaccggguacgcauucUuccg....................   1    1   Mm1
......................................................................ucaccggguacgcauucauccU....................   1    1   Mm1
......................................................................ucaccggguacgcauucauccg....................   82   0   Mm1
......................................................................ucaccggguacgcauucGuccg....................   1    1   Mm1
......................................................................Ccaccggguacgcauucauccg....................   1    1   Mm1
......................................................................ucaccggguacgcauucauccC....................   8    1   Mm1
......................................................................ucaccggguacgcauucauccgg...................   13   0   Mm1

.................................ggugaauacgaacccagguuaug........................................................   4    0   Mm2
......................................................................ucaccggguacgcauucau.......................   1    0   Mm2
......................................................................ucaccggguacgcauucauc......................   35   0   Mm2
......................................................................ucaccggguacgcauucGucc.....................   1    1   Mm2
......................................................................ucacUggguacgcauucaucc.....................   1    1   Mm2
......................................................................ucaccggguacgcauucaucU.....................   1    1   Mm2
......................................................................ucaccggguacgcauuUaucc.....................   1    1   Mm2
......................................................................ucaccgAguacgcauucaucc.....................   1    1   Mm2
......................................................................ucaccggguacgcauucaucc.....................   77   0   Mm2
......................................................................ucaccggguacgcauucCuccg....................   2    1   Mm2
......................................................................uUaccggguacgcauucauccg....................   1    1   Mm2
......................................................................ucaccggguacgcauuUauccg....................   1    1   Mm2
......................................................................ucaccggguacgcauucauccU....................   13   1   Mm2
......................................................................ucaccggguacgcauucaucUg....................   1    1   Mm2
......................................................................ucaccggguCcgcauucauccg....................   1    1   Mm2
......................................................................ucaccggguacgcauucauccg....................   156  0   Mm2
......................................................................ucaccggguaGgcauucauccg....................   1    1   Mm2
......................................................................ucaccggguacgcauuAauccgg...................   1    1   Mm2
......................................................................ucaccggguacgcauucUuccgg...................   1    1   Mm2
......................................................................ucaccggguacgcauucauccgU...................   14   1   Mm2
......................................................................ucaccggguacgcauucauccgg...................   51   0   Mm2
......................................................................ucaccggguacgcauucauccgA...................   1    1   Mm2
......................................................................ucaccggguacgcauucauccggU..................   1    1   Mm2
```
